# Identification and characterization of the bacterial etiology of clinically problematic acute otitis media after tympanocentesis or spontaneous otorrhea in German children

**DOI:** 10.1186/1471-2334-12-312

**Published:** 2012-11-20

**Authors:** Gerhard Grevers, Susanne Wiedemann, Jan-Christof Bohn, Rolf-Werner Blasius, Thomas Harder, Werner Kroeniger, Volker Vetter, Jean-Yves Pirçon, Cinzia Marano

**Affiliations:** 1 ENT Center, Prinzenweg 1, 82319, Starnberg, Germany; 2 Am Plärrer 25, Nürnberg, Germany; 3 ENT Center, Markt 18, 09648, Mittweida, Germany; 4 ENT Center, Kaiserstrasse 8, 33790, Halle, Germany; 5 Skandinaviendamm 251, 24109, Kiel, Germany; 6 GlaxoSmithKline GmbH & Co. KG, Munich, Germany; 7 GlaxoSmithKline Vaccines, Wavre, Belgium

**Keywords:** otitis media, *Streptococcus pneumoniae*, *Haemophilus influenzae*, Tympanocentesis, Antibiotic resistance

## Abstract

**Background:**

Acute Otitis Media (AOM) is an important and common disease of childhood. Bacteria isolated from cases of clinically problematic AOM in German children were identified and characterized.

**Methods:**

In a prospective non-interventional study in German children between 3 months and less than 60 months of age with Ear, Nose and Throat Specialist –confirmed AOM, middle ear fluid was obtained by tympanocentesis (when clinically indicated) or by careful sampling of otorrhea through/at an existing perforation.

**Results:**

In 100 children with severe AOM, *Haemophilus influenzae* was identified in 21% (18/21, 85.7% were non-typeable [NTHi]), *Streptococcus pneumoniae* in 10%, *S. pyogenes* in 13% and *Moraxella catarrhalis* in 1%. *H. influenzae* was the most frequently identified pathogen in children from 12 months of age. *H. influenzae* and *S. pneumoniae* were equally prevalent in children aged 3–11 months, but *S. pyogenes* was most frequently isolated in this age group. NTHi AOM disease appeared prevalent in all ages.

**Conclusions:**

NTHi, *S. pneumoniae* and *S. pyogenes* are implicated as important causes of complicated AOM in children in Germany. NTHi disease appears prevalent in all ages. The impact of vaccination to prevent NTHi and *S. pneumoniae* AOM may be substantial in this population and is worth investigating.

## Background

Acute Otitis Media (AOM) is one of the most frequently reported childhood diseases and is the primary reason for antibiotic use in children [1]. Globally, the major bacterial pathogens responsible for AOM are *Streptococcus pneumoniae, Haemophilus influenzae* and to a lesser extent, *Moraxella catarrhalis*, *S. pyogenes* and *Staphylococcus aureus*[2]*.* Little is known about AOM microbial epidemiology in Germany. Perforation of the tympanic membrane (TM) can occur as a consequence of AOM. This has potentially important long term clinical consequences [1].

Widespread vaccination with 7-valent pneumococcal conjugate vaccine (PCV7) has reduced outpatient visits due to AOM in children <2 years of age in the United States, although the magnitude of the effect on AOM incidence remains unclear [3]. PCV7 vaccination has also been linked to an overall reduction in AOM cases due to vaccine related *S. pneumoniae*, and a relative increase in cases due to *H. influenzae* and non-vaccine *S. pneumoniae* serotypes [4].

PCV7 was introduced to the German market in 2001 and became universally recommended for all infants in 2006. No available data from Germany has examined pathogen distribution in AOM using direct Middle Ear Fluid (MEF) sampling [5,6]. The aim of this prospective non-interventional study was to assess the bacterial etiology and antimicrobial susceptibility of bacteria causing AOM in 100 German children between 3 months and less than 60 months of age. In Germany, tympanocentesis is only indicated for treatment of complications of AOM, treatment failure and in conditions of imminent TM perforation to avoid spontaneous perforation [7]. In our study, MEF samples were collected from spontaneous TM ruptures by an Ear, Nose and Throat (ENT) specialist as part of routine clinical ENT practice or when tympanocentesis was clinically indicated.

The information from this study is important for development and refinement of AOM treatment guidelines, and for assessing potential benefits of new PCVs.

## Methods

The study (111640) was conducted in 11 centers in Hamburg, Münster, Berlin, Dresden, München, and Bad Segeberg in Germany. All children between 3 months and less than 60 months of age with ENT specialist–confirmed AOM were assessed for eligibility by the ENT specialist at the study center. Written informed consent to analyze MEF samples was obtained from the parents/guardians of eligible children. The treatment of each child was left to the discretion of the ENT specialist. The study protocol was approved by the local ethics committees: the Ethik-Kommission bei der Ärztekammer Hamburg, Ethik- Kommission der Medizinischen Fakultät der Universität Münster, Ethik-Kommission der Sächsischen Landesärztekammer, Ethik-Kommission der Bayerischen Landesärztekammer, Ethik-Kommission der Ärztekammer Schleswig-Holstein and the Ethik-Kommission der Ärztekammer Berlin. MEF samples were collected by the ENT specialist from spontaneous TM ruptures as part of routine clinical ENT practice or when tympanocentesis was clinically indicated.

### Eligibility criteria

AOM diagnostic criteria were:1) acute onset of signs and symptoms including fever, otalgia, irritability; 2) the presence of MEF; and 3) recent onset (within 72 hours) of signs and symptoms of middle-ear inflammation. Children were eligible if they had a diagnosis of AOM; and if a MEF sample had been obtained; and if they had either not been previously treated with antibiotics, or if they had treatment failure (no clinical improvement within 48– to 72 hours of antibiotic treatment).

Children were not eligible if the following conditions applied: if they been hospitalized for more than 48 hours prior to the diagnosis of AOM perforation; if they had otitis externa or non-acute otitis media with effusion; if otorrhea was present for more than 48 hours prior to sampling; if they had a trans-tympanic aerator *in situ;* if they had received systemic antibiotic treatment for other conditions 48 to 72 hours prior to study entry; or if they had received prophylactic antibiotics for recurrent AOM. Each child contributed one AOM episode to the study.

Recurrent AOM was defined as the third or greater new episode within the past 6 months, or the fourth or greater new episode within the past year.

### Study procedures

MEF was obtained by tympanocentesis or by careful sampling of otorrhea through an existing perforation as a routine procedure performed by the ENT specialist. These procedures were performed under microscopic visualization following removal of debris and cleaning of the external auditory canal. Samples were inoculated onto Amies transport medium (Copan Innovation transystem Amies without carbon Reference 190C) and sent at room temperature via courier to the National Reference Laboratory (analysis managed by MvdL) within 16 hours (maximum 72 hours). Initially transported samples were partially frozen and the delivery services were subsequently changed. Samples were inoculated onto chocolate agar (otorrhea samples were inoculated onto chocolate agar with bacitracin), blood (with gentamycin) and MacConkey agar. Identification of *S. pneumoniae*, *H. influenzae*, *M. catarrhalis*, and *S. pyogenes* was by standard bacteriological procedures [8]. *S. pneumoniae* serotypes were identified by Quellung reaction. *H. influenzae* serotypes were identified using monovalent anti-sera. Mean inhibitory concentrations (MIC) were determined by microdilution tests interpreted according to National Committee on Clinical Laboratory Standards guidelines.

## Results

### Clinical and demographic characteristics of enrolled subjects

One hundred and twelve children with AOM were recruited between November 2008 and April 2010. Twelve children did not meet the inclusion/criteria and were eliminated from the According-to-protocol (ATP) cohort. Three episodes were treatment failures (defined as no clinical improvement within 48–72 hours of antibiotic treatment) and the remaining 97 cases had not been previously treated with antibiotics. The mean age of children was 35.2 months (range 3 to 59 months) and 44.0% were female. Eight percent of the cases were in the age range 3 to 11 months, 15% were in the 12 to 23 month range, 26% were in the 24 to 35 month range, 21% in the 36 to 47 month range, and 30% in the 48 to 59 month age range.

The majority (74%) children had received at least one PCV7 dose. Clinical symptoms such as fever >39.0°C, ear discharge, lethargy, irritability and anorexia were reported more frequently among children with cultures positive for *S. pneumoniae* compared to children with cultures positive for *H. influenzae*.

There were 23 recurrent AOM cases (23% [95% confidence interval CI: 15–32]). A total of 25% [95% CI: 3–65] of cases were recurrent in 3–11 month olds, 27% [95% CI: 8–55] in 12–23 month olds, 31% [95% CI: 14–52] in 24–35 months, 19% [95% CI: 5–42] in 36–47 months and 17% [95% CI: 6–35] in 48–59 month olds.

### Microbiological results

One sample was collected from each child (otorrhea in 76 cases and MEF via tympanocentesis in 24 cases). In each age group, the majority of samples were otorrhoea (between 71% and 88% of all samples).

Of 100 samples, 44% (44/100) were culture positive for one or more of the four bacteria under study: 21 (39.6%) for *H. influenzae*, 10 (18.9%) for *S. pneumoniae*, 13 (24.5%) for *S. pyogenes* and 1 (2%) for *M*. *catarrhalis*. Other bacteria including *Pseudomonas aeruginosa* and *S. aureus,* were identified in 15 cases (Table 1). Both episodes positive for *P. aeruginosa* and all but one of 8 episodes positive for *S. aureus* were from otorrhoea samples, possibly reflecting flora of the external auditory canal.

**Table 1:** Bacterial etiology of episodes (ATP cohort)

	**Otorrhoea N = 76**		**Tympanocentesis N = 24**		**Total N = 100**	
**Culture positive for**	**n**	**%**	**n**	**%**	**n**	**%**
Any growth	43	57	10	42	53	53
*Streptococcus pneumoniae*	10	13	0	0	10	10
*Haemophilus influenzae*	14	18	7	29	21	21
*Streptococcus pyogenes*	13	17	0	0	13	13
*Moraxella catarrhalis*	0	0	1	4	1	1
*Achromobacter spp.*	1	8	0	0	1	7
*Acinetobacter lwoffii*	0	-	1	50	1	7
*Coryneobacertium spp.*	1	8	0	0	1	7
*Escherichia coli*	1	8	0	0	1	7
*Pseudomonas aeruginosa*	2	15	0	0	2	13
*Staphylococcus aureus*	8	62	1	50	9	60

N = number of episodes.

% = n/Number of episodes with available results x 100.

Note: More than one other bacteria was detected for some samples.

Cultures were positive in 8/24 tympanocentesis samples (33%) and 36/76 otorrhea samples (47%). *H. influenzae* was identified in 18% of otorrhea samples and 29% of tympanocentesis samples. *S. pneumoniae* and *S. pyogenes* were isolated in 13% and 17% of otorrhea samples, respectively. No tympanocentesis samples were positive for *S. pneumoniae* or *S. pyogenes*.

**Figure 1:**
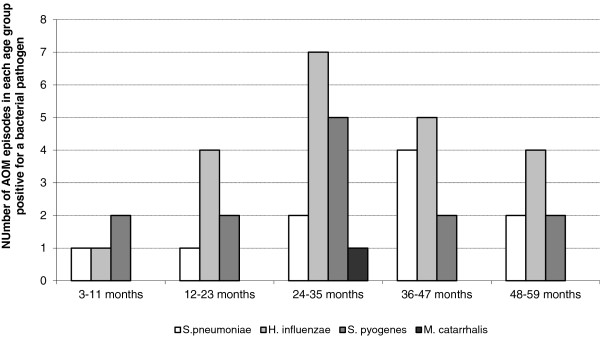
Bacterial etiology of 100 AOM episodes by age (ATP cohort).

*H. influenzae* was the most frequently identified pathogen in all age groups from 12 months of age (Figure 1): 18/21 (85.7%) *H*. *influenzae* isolates were non-typeable, one was type b and one was type f. Of 10 *S. pneumoniae* isolates, 6 were serotype 3, 3 were serotype 19A and 1 was serotype 1.

The distribution of pathogens was similar in PCV7-vaccinated and unvaccinated subjects. For *H, influenzae*, 52% (11/21) of cases occurred in children who had received 4 PCV7 doses.

Most (9/10, 90%) *S. pneumoniae* isolates were penicillin sensitive and all were susceptible to other antibiotics, including macrolides. Among Hi isolates, resistance to trimethoprim/sulfamethoxazole was observed in 33% (7/21), resistance to ampicillin in 19% (4/21) and resistance to amoxicillin in 10% (2/21). None of the ampicillin resistant strains were β-lactamase negative ampicillin resistant strains.

## Discussion

In this prospective study of AOM characteristics in Germany, *H. Influenzae* was the most frequently identified pathogen in children 12 months of age and older, and was also frequently identified in 3–11 month olds, consistent with a growing body of evidence implicating *H. Influenzae* as a key pathogen in AOM [2,4], and of a possibly increasing role due to PCV vaccination. *S. pyogenes* was the second most frequently identified pathogen. These results are consistent with preliminary results from another study conducted in Germany [6], but contrast with other studies suggesting a possibly diminishing role for *S. pyogenes* in AOM etiology [2]. In a large evaluation of AOM etiology conducted in Israel, *S. pyogene*s AOM was observed more frequently in older children than in younger children, and was often associated with acute TM perforation [9]. In our study *S. pyogenes* was only isolated from otorrhea samples (not from tympanocentesis samples), supporting the notion that *S. pyogenes* infection rapidly leads to TM perforation [9]. The high rate of *S.pyogene*s isolation in the present study likely reflects the severity of disease in the enrolled population (children with TM perforation or with clinically indicated tympanocentesis according to German clinical guidelines).

In the present cohort at least 74% of children had received at least one PCV7 dose. Although the low number of subjects precludes firm conclusions, the results are generally consistent with larger observational studies showing an increase in AOM due to non-PCV7 *S. pneumoniae* serotypes in countries where PCV7 uptake is high [4]. New pneumococcal conjugate vaccines targeting *H. influenzae* by use of Protein D as carrier protein (PHiD-CV) [10], may provide additional benefits in Germany.

In contrast with other countries in Europe, antibiotic susceptibility rates were high in German children. This may be due in part to reduced penicillin consumption in Germany, which is lower than in many other countries in Europe [11].

The potential limitations of the study include the low number of culture-positive samples for which a cause was not able to be identified, although the percentage of positive cultures increased (from 43% to 61%) after changes were made to ensure that sample delivery times and temperature constraints were met. It is also possible that the initial delay between sample collection and processing might have introduced a bias toward a differential recovery of more resilient organisms. For example, *S. aureus* and *S. pyogenes* are known to overgrow other bacteria on swabs when processing delay increases, and both *S. pneumoniae* and NTHi are fastidious organisms more likely to perish after 6 hours and in conditions of freezing.

Because tympanocentesis is not routinely performed for AOM management in Germany, only children with spontaneous perforations in which a specimen was obtained using a procedure avoiding contamination or those in whom tympanocentesis was clinically indicated were eligible. Thus, our population had more severe AOM and the results may not necessarily be able to be extrapolated to all cases of AOM.

## Conclusions

In the present study, NTHi, *S. pneumoniae* and *S. pyogenes* were implicated as important causes of complicated AOM in children in Germany. NTHi disease appeared prevalent in all ages. The impact of vaccination to prevent both *S. pneumoniae* and NTHi AOM may be substantial in this population and is worth investigating.

## Abbreviations

AOM: Acute otitis media; ATP: According to protocol; CI: Confidence interval; ENT: Ear, nose and throat specialist; MEF: Middle ear fluid; PCV7: 7-valent pneumococcal conjugate vaccine; TM: Tympanic membrane.

## Competing interests

GG has received consulting fees and support for travel to scientific meetings from GlaxoSmithKline Biologicals SA.

SW, JCB, TH and RWB have received consulting fees from GlaxoSmithKline Biologicals SA.

JYP, WK, VV and CM are employees of GlaxoSmithKline. Biologicals SA. VV, WK and CM declare ownership of stock options in GlaxoSmithKline

## Authors’ contributions

GG helped design and plan the study, collected data and was involved in interpretation of the results. SW, JCB, RWB and TH collected the data and were involved in interpretation of the results. VK and VV helped plan the study and were involved in interpretation of the results. JYP helped design the study, performed the statistical analyses and was involved in interpretation of the results. CM helped design and plan the study, assisted with the analysis and with interpretation of the results. All authors critically reviewed each draft of the manuscript and approved the final version.

## Pre-publication history

The pre-publication history for this paper can be accessed here:

http://www.biomedcentral.com/1471-2334/12/312/prepub
