# Erratum to: ‘Identification and characterization of the bacterial etiology of clinically problematic acute otitis media after tympanocentesis or spontaneous otorrhea in German children’

**DOI:** 10.1186/s12879-016-1415-4

**Published:** 2016-03-09

**Authors:** Gerhard Grevers, Susanne Wiedemann, Jan-Christof Bohn, Rolf-Werner Blasius, Thomas Harder, Werner Kroeniger, Volker Vetter, Jean-Yves Pirçon, Cinzia Marano

**Affiliations:** ENT Center, Prinzenweg 1, 82319 Starnberg, Germany; Am Plärrer 25, Nürnberg, Germany; ENT Center, Markt 18, 09648 Mittweida, Germany; ENT Center, Kaiserstrasse 8, 33790 Halle, Germany; Skandinaviendamm 251, 24109 Kiel, Germany; GlaxoSmithKline GmbH & Co. KG, Munich, Germany; GlaxoSmithKline Vaccines, Wavre, Belgium

Unfortunately, the original version of this article [1] contained missing infromation. In the study procedures it was mentioned that samples were to be inoculated onto chocolate agar (otorrhea samples were inoculated onto chocolate agar with bacitracin), blood (with gentamycin) and MacConkey agar. Instead, the samples have been routinely plated on Columbia agar with sheep blood, colistin/nalidixin columbia (CNA) agar with sheep blood, McConkey agar, supplemented chocolate agar and Sabouraud agar. The use of the different agar does not have any impact on the study results and conclusions. We express our gratitude to Mark van der Linden and Matthias Imöhl from the German National Reference Center for Streptococci, who performed all microbiological analyses.

## References

[1] Grevers G, Widemann S, Bohn JC. Identification and characterization of the bacterial etiology of clinically problematic acute otitis media after tympanocentesis or spontaneous otorrhea in German children. 2012;12:312. 10.1186/1471-2334-12-312 PMC3544702 23167692

